# A childhood obesity intervention developed by families for families: results from a pilot study

**DOI:** 10.1186/1479-5868-10-3

**Published:** 2013-01-05

**Authors:** Kirsten K Davison, Janine M Jurkowski, Kaigang Li, Sibylle Kranz, Hal A Lawson

**Affiliations:** 1 Department of Nutrition, Harvard School of Public Health, Harvard University, Boston, MA, USA; 2 Department of Health Policy, Management and Behavior, School of Public Health, University at Albany, Albany, NY, USA; 3 Prevention Research Branch, National Institute of Child Health & Human Development, Bethesda, MD, USA; 4 Department of Nutrition Sciences, College of Health and Human Services, Purdue University, West Lafayette, IL, USA; 5 School of Social Welfare, University at Albany, Albany, NY, USA

**Keywords:** Community-based participatory research, CBPR, Action research, Head Start, Diet, Physical activity, Family intervention

## Abstract

**Background:**

Ineffective family interventions for the prevention of childhood obesity have, in part, been attributed to the challenges of reaching and engaging parents. With a particular focus on parent engagement, this study utilized community-based participatory research to develop and pilot test a family-centered intervention for low-income families with preschool-aged children enrolled in Head Start.

**Methods:**

During year 1 (2009–2010), parents played an active and equal role with the research team in planning and conducting a community assessment and using the results to design a family-centered childhood obesity intervention. During year 2 (2010–2011), parents played a leading role in implementing the intervention and worked with the research team to evaluate its results using a pre-post cohort design. Intervention components included: (1) revisions to letters sent home to families reporting child body mass index (BMI); (2) a communication campaign to raise parents’ awareness of their child’s weight status; (3) the integration of nutrition counseling into Head Start family engagement activities; and (4) a 6-week parent-led program to strengthen parents’ communication skills, conflict resolution, resource-related empowerment for healthy lifestyles, social networks, and media literacy. A total of 423 children ages 2–5 years, from five Head Start centers in upstate New York, and their families were exposed to the intervention and 154 families participated in its evaluation. Child outcome measures included BMI z-score, accelerometer-assessed physical activity, and dietary intake assessed using 24-hour recall. Parent outcomes included food-, physical activity- and media-related parenting practices and attitudes.

**Results:**

Compared with pre intervention, children at post intervention exhibited significant improvements in their rate of obesity, light physical activity, daily TV viewing, and dietary intake (energy and macronutrient intake). Trends were observed for BMI z-score, sedentary activity and moderate activity. Parents at post intervention reported significantly greater self-efficacy to promote healthy eating in children and increased support for children’s physical activity. Dose effects were observed for most outcomes.

**Conclusions:**

Empowering parents to play an equal role in intervention design and implementation is a promising approach to family-centered obesity prevention and merits further testing in a larger trial with a rigorous research design.

## Background

Once limited to children 4 years and older [1], epidemic rates of obesity are now evident in very young children. Today, 1 in 10 infants and 1 in 4 toddlers and preschool-aged children are overweight or obese [2]. The public health burden of obesity is extreme given its immediate and long term health consequences [3] and its ultimate effect on life expectancy [4]. This burden is disproportionately experienced by children from low-income and ethnic minority families and serves to perpetuate health disparities [5,6].

Given the essential and pivotal role that families play in shaping children’s early life experiences, the prevention of obesity in young children will require effective approaches for working with families, starting with parents and caregivers. Family-centered interventions focus on the needs of children and adolescents while simultaneously targeting improved outcomes for the entire family system [7]. What is more, family-centered interventions emphasize intra-familial and contextual factors that define and govern daily life and family decision making [7]. While the need to focus on families is increasingly recognized as an important strategy to address childhood obesity, surprisingly few programs are family-centered [8,9]. Family engagement is typically indirect, through newsletters and family fun nights, and family involvement is usually a minor component of a larger intervention [10-12]. Furthermore, family dropout rates are high (i.e., 27-73%) [13], with the highest rates observed among the most vulnerable families [13].

The paucity of family-centered interventions for childhood obesity may be explained, in part, by researchers’ and service providers’ uncertainty about how to engage family members, especially vulnerable parents, in interventions and their evaluation [11,12,14]. For example, although researchers have sought parental input during formative stages of program development, parents have had little decisional control over the resulting interventions. The ensuing interventions do not take into account family realities, are often poorly attended, [13] and lack sustained impact, [10,14].

In this study, we present a new approach to family-centered childhood obesity prevention. In contrast to the typical approach of indirect parent engagement, the Communities for Healthy Living (CHL) program was developed in collaboration with low-income parents and caregivers (referred to hereafter as parents) of preschool-aged children and representatives from community organizations. A community-based participatory research (CBPR) approach was utilized to ensure that parents and community-based organizations were actively engaged in the design, implementation and evaluation of the program. CBPR is growing in value and federal investment [15]. It is an effective strategy for gaining local knowledge of sociocultural contexts for the development of culturally-tailored interventions. A key tenet of CBPR is the identification of assets and facilitators of health [16]. Resulting interventions are more likely to leverage individual, institutional, and community assets and provide salient knowledge, skills and resources to the target population than traditional deficit-focused models [16]. This in turn fosters the sustainability of a program.

While CBPR is increasingly utilized in health initiatives, health and human service professionals and community leaders are predominantly engaged as representatives of the community served. Parents, particularly those from low-income backgrounds, are rarely viewed and leveraged as experts. In this study, we introduce *a parent-centered CBPR approach* for obesity prevention in vulnerable families. Our intervention is designed from the ground up with parents as the majority of the decision making body. Using a pre-post cohort design, we test its initial efficacy for improving food, physical activity and media-related parenting and children’s behavioral and weight outcomes.

## Methods

### Theoretical foundation

The overarching design of this study was guided by the Family-centered Action Model of Intervention Layout and Implementation (FAMILI) [7] and its interdisciplinary foundations in nutrition, child development, and public health. FAMILI emphasizes the need to (a) draw on theories of child and family development, (b) utilize mixed methods to capture and understand the lived experiences of families, and (c) actively engage families in interventions from program development through evaluation.

While FAMILI provides the overarching foundation for the study, specific subcomponents of the study are informed by the Family Ecological Model (FEM) – a family-centered developmental theory – and Empowerment Theory. The FEM postulates that caregiving practices and family daily living strategies are shaped by factors proximal to families (e.g., child characteristics, family history and structure, and family health) in combination with the broader contexts. Broader contexts can encompass parent job characteristics and demands, school policies, community food and activity resources, and neighborhood social capital [17,18]. The implication is that effective family-centered interventions will require deep understanding of the contexts in which families are embedded.

According to the Empowerment Theory guiding this study, empowerment results from understanding the forces that affect life situations (i.e., critical consciousness) combined with the ability to control these forces using resources and social supports gained through social capital networks [19]. Empowerment is fostered through critical reflection andequitable collaboration and results in increased power and resource redistribution [19,20]. Based on this hybrid empowerment model, we predicted that parents’ active participation in the research process would foster critical reflection on the real life issues that contribute to obesity in their community while helping parents identify and then address in their decision-making important social, cultural and environmental factors that contribute to healthy lifestyles. Together these mechanisms were hypothesized to result in a family-centered program that was culturally responsive and effective—as indicated by measurable outcomes.

### Setting

The CHL program was developed and pilot-tested in five Head Start centers serving 423. 2–5 year old children in upstate New York. Head Start, a US federal program promoting health and school readiness in low-income preschool-aged children [21], was selected as the focal setting for this study because parent involvement is a core component of its mission. Also, in recognition of the conflicting demands placed on vulnerable families, Head Start provided the opportunity to embed the process into a system of care [22]. Consistent with the demographics of upstate New York, 38.5% of children enrolled in the centers were non-Hispanic White, 17.8% were non-Hispanic Black, 13.5% were biracial, 6.1% were Hispanic or Latino, and 24% did not have race/ethnicity documented. Approximately, 90% of households reported speaking English, and 6% reported speaking Spanish, as their primary language.

### Intervention development and implementation

Development of the CHL program took place over a one-year period between Sept 2009 and August 2010. Formation of CHL’s community advisory board (CAB) during the first two months of the project was the foundation of the participatory process. Parents of Head Start children, who comprised the majority of the CAB, were recruited through the Head Start Policy Council and word of mouth by organizational staff, the Policy Council parents, and the project coordinator. Community organization representatives (e.g., a large pediatric provider serving predominantly low-income children and a reverend from a local church) and key agency Head Start staff were also invited to form the CAB. The organizational members were recruited based on recommendations and their work with low income families in the focal community.

A total of 20 CAB members were recruited, 17 of whom participated beyond the first meeting. CAB meetings were held 1–2 times per month during the first six months of the project, culminating in a community assessment, and were held monthly thereafter. A total of 25 CAB meetings were held over the course of the two year study. CAB meetings were led by the second author, an expert in CBPR. The parent engagement process, which is briefly summarized below, is outlined in detail in a published case study [23].

### The process of engaging parents

The CAB developed and approved partnership principles to provide guiding values and codified expectations and operating guidelines to sustain active involvement. To operationalize a participatory process, various strategies and structural accommodations were employed to foster parents’ involvement throughout all phases of the research process. First, CHL emphasized from the beginning that parents were ‘experts’ with unique knowledge and experiences about parenting and the family context. They were equated to professional and research experts. All strategies and activities were developed with this frame of reference, which set a tone that remained throughout the project.

Second, structural factors weighed heavily in the participatory process, including meeting in the community and providing compensation, meals, and child care. The CAB was also split into four small workgroups that worked on multiple components of the research simultaneously. CAB members were involved in as many activities as they were willing to participate. In addition to participating in CAB meetings, parents participated in day to day research activities alongside the researchers as equal partners [23].

Finally, the CAB was considered an intervention in of itself with its own evaluation [24]. This led to theory-guided activities and reflection. For example, CAB discussions and break-out sessions were guided by the FEM [18]; CAB meetings focused on personal definitions of health, strategies used to foster family health, chronic stressors that affected parenting and family interactions, links between communities and families, and resources families drew on to support health. Findings from these discussions were instrumental in the development of a multi-method community assessment to examine factors of greatest interest to the CAB members and the Head Start families they represented.

### Conducting a community assessment

Multiple methods of assessment were utilized in the community assessment including self-report surveys, focus groups, Photovoice, [25] and windshield surveys [26]. Children’s weight status, dietary intake and physical activity were also measured. Survey questions examined the roles of parents and older children in the household, family utilization of community programs and services, and parents’ viewpoints on childhood obesity. Focus groups examined the impact of having children over a wide age range on food, physical activity and screen-related parenting. For the Photovoice protocol, parents documented by camera the chronic and acute stressors they experienced over the course of their day. For the windshield surveys, parents were led on a driving tour of their neighborhood and answered open-ended questions about the perceived social, economic and environmental conditions of their neighborhood and their effect on their daily activities, parenting, and children’s well-being. Additional information on the community assessment, and a summary of the results, are described in detail elsewhere [18].

In addition to defining the scope of the community assessment, CAB members were invited to participate in the collection and interpretation of the data. Results from the community assessment were shared in two community forums with the CAB, the broader community of Head Start parents, community members, and Head Start staff and teachers. The final CHL program was developed utilizing results from the community assessment, feedback obtained during the forums, and subsequent discussions with the CAB. Primary objectives of the program were to (1) promote parenting practices supportive of healthy lifestyles (e.g., limiting children’s screen time, encouraging consumption of fruits and vegetables, promoting outdoor play), (2) increase children’s healthy lifestyle behaviors (e.g., improved diet, increased physical activity, and decreased television viewing time), and (3) reduce children’s BMI and rates of obesity. The program was pilot-tested during this project’s second year.

### Intervention components

Summarized in Table 1, the CHL program comprised four key components. First, a health communication campaign, which integrated quotes from the focus groups conducted during the community assessment, was developed to increase parents’ awareness of childhood obesity and dispel myths around children’s weight (e.g. “it’s just baby fat, he will grow out of it”) [27]. Second, letters mailed home to families by Head Start reporting children’s BMI , and other health indicators, were revised based on parent feedback to facilitate parents’ understanding of the information provided. Third, informal nutritional counseling sessions were integrated into Head Start family engagement activities. Community nutrition graduate students from a local college attended Head Start family events, provided samples of healthy foods, and were available to answer questions parents had about their child’s (or their own) diet and weight status.

**Table 1:** Summary of the Communities for Healthy Living (CHL) Intervention

**Intervention component**	**Description**	**Intervention principles**	**Link with community assessment findings **[1]
Health Communication Campaign	Posters (N=6) displayed on a rotating basis in all Head Start centers for 3–4 weeks each. Each poster was also sent home as a flyer with information about other components of the CHL program printed on the back.	·Increase parent awareness and recognition of their child’s weight status	·Parents displayed low awareness of childhood obesity and its health ramifications
			·Parents endorsed myths about obesity
		·Dispel myths about children’s weight status (e.g., he’s just big for his age, my child is active she can’t be overweight, juice is good for my child)	
Revised Body Mass Index (BMI) letters	Letters sent home to families with results from their child’s height and weight measurements were revised to improve the accessibility of information for parents. Additional information outlined how to interpret child BMI and weight status and identify community resources to prevent/treat overweight in children	·Increase parent awareness and understanding of child weight status	·Parents displayed low awareness of childhood obesity
			·Parents reported that they did not understand the content of the BMI letters sent home by Head Start
		·Increase parent awareness of local resources for obesity prevention and treatment	
Family nutrition counseling	Informal nutrition counseling sessions were integrated into Head Start family engagement activities. Local nutrition graduate students attended Head Start family events, provided samples of healthy foods and answered any questions parents had regarding their child’s and their own nutrition and weight status,.	·Foster parent social networking	·Parents reported an interest in connecting with other Head Start parents and sharing information.
		·Promote parent resource empowerment	
			·Few services for childhood weight management were available in the community.
		·Increase parent nutrition knowledge	
Parents’ Connect for Healthy Living Program	Six weekly 2-hour sessions implemented in each Head Start center. All sessions addressed skills that parents were most interested in gaining, incorporated materials/examples around healthy living, and included workshops by local organizations (e.g., media literacy training provided by a local public broadcasting station). Sessions were led by trained parent leaders in conjunction with an experienced group moderator.	Sessions included materials/examples specific to healthy living and addressed the following:	·Parents expressed an interest in developing the skills outlined during the community assessment.
		·Resource identification and utilization	·Children watched extensive amounts of TV. Parents reported high levels of stress and a need to rely on child screen time as down time or to get things done. Media literacy training was intended to support parents in making mindful decisions about child screen time (i.e., to make active decisions about when and what a child could watch).
		·Effective communication	
		·Conflict resolution	
		·Media literacy	
		·Professionalism	
Child program	Held concurrently with the parent program for children accompanying their parents. Engaged children in activities similar to the parent program. Mini workshops were run by local organizations (e.g., dance studios, karate)	·Enjoyment of active recreation	
		·Media literacy	

[1] Community assessment findings are summarized in Davison, Jurkowski & Lawson (in press). Family-centered obesity prevention redefined: The Family Ecological Model. *Public Health Nutrition*.

The final and central component of CHL was the Parents Connect for Healthy Living program, a 6-week, onsite, parent-led program to promote parent social networking, advocacy, communication skills, media literacy and conflict resolution-all of which were behavioral targets of interest to parents identified through the community assessment [18]. Parent leaders, in collaboration with an experienced group moderator, led group sessions. In preparation for this role, parent leaders completed a two-day intensive training session. Workshops, led by local organizations, were integrated into the weekly sessions. For example, the local public broadcasting station led a workshop on media literacy in an effort to encourage parent regulation of child screen time. A child program was held concurrently to the parent program for accompanying children. In addition to crafts and other activities, children participated in workshop sessions led by the same community organizations separately from parents as feasible.

The program was implemented in Head Start centers over a six-month period between November 2010 and April 2011. During program implementation, the health communication campaign was implemented over a 3-month period (January-March 2011), the revised BMI letters were sent home twice to families (in fall and spring), eight family nutritional counseling sessions were held in centers, and the 6-week Parents’ Connect Program and associated child program were implemented twice (in fall and spring).

### Evaluation design

A pre-post cohort design was used to evaluate the CHL program. All families with a child 2 years or older enrolled in the target Head Start centers were eligible to participate in the evaluation. Families were recruited through posters displayed in centers and flyers sent home with children. Baseline data were collected between September and November 2010. Follow-up data were collected between April 2011 and June 2011. Process-related questions, including program exposure, were included on the follow-up survey.

By consenting to participate in the study, parents agreed to complete a self-report survey at baseline and follow-up and gave permission for the investigators to extract their child’s BMI data from Head Start records. Parents received a $20 gift card at baseline and follow-up for successful completion of these activities. A total of 154 parents completed this protocol at baseline, 119 of whom also participated at follow-up (reflecting 77% retention). Parents could also provide separate consent for the 24-hour dietary recall procedure and the accelerometry protocol. Families were compensated with a $20 gift card for each procedure at baseline and $30 at follow-up. At baseline, a total of 55 parents completed the dietary recall procedure and 83 children completed the accelerometry protocol, of whom 33 parents and 57 parents completed the dietary recall and accelereometry protocols respectively at follow-up.

### Measures

#### Child weight status

Data on children’s height and weight were obtained from Head Start records in fall 2010 (prior to intervention implementation) and again in spring 2011. To ensure accurate measurement, children’s height and weight were measured at each time point by trained research staff in conjunction with Head Start personnel. Additionally, research-quality calibrated scales and portable stadiometers, provided by the research team, were utilized. The resulting data were entered into the Head Start database according to the usual protocol. BMI z-scores and weight status classification were then extracted for children whose parents provided written consent. Children’s age- and sex-specific BMI percentiles and z-scores were calculated using CDC 2000 growth charts. BMI percentile scores were used to identify children who were overweight (85^th^ – 94.99^th^ BMI percentile) or obese (95^th^ or higher BMI percentile) [28].

#### Child dietary intake

Children’s dietary intake was estimated using two 24-hour dietary recalls with mothers reporting children’s diets as proxies [29]. At pre and post intervention, two recalls were obtained within a 10-day period including one weekday and one weekend recall. All recalls were conducted by phone by trained staff at Purdue University. Interviewer training included a standardized mock recall to determine the intra-rater reliability; a deviation of plus/minus 5% of total energy intake was estimated as acceptable. All recalls followed the standardized protocol of the Nutrient Data System for Research (NDSR) program [29] and used multiple pass 24-h methodology [30]. At pre and post intervention, dietary intake data were averaged across the two days to estimate average daily energy intake (kcal), macronutrient intake (in grams), and food group servings.

#### Child physical activity (PA)

Children’s physical activity and inactivity were measured using a GT3X accelerometer, worn around the waist for 7 days. The monitors were initialized to record data in 15s intervals or epochs [31,32]. Children with at least 4 days of monitoring data, for a minimum of 10 hours per day, were considered compliant and included in the analyses [33,34]. Minutes per hour spent in moderate PA (MPA), light PA and sedentary activity were calculated using age-appropriate cut-points. [35] Of the 117 children whose parents provided consent for them to wear the activity monitor, 83 met the criteria of compliance and included in the analyses of child PA.

#### Child TV-viewing

As a measure of children’s TV viewing time, parents indicated how much time (hours and minutes) their child spent watching TV, DVDs or videos on a typical (a) weekday and (b) weekend day; responses were coded to reflect average viewing time per day.

#### Parenting for healthy lifestyles

A self-report survey was used to measure parenting practices and attitudes specific to children’s physical activity, diet, and screen time.

Eight items from the Activity Support Scale [36] were used to measure physical activity and screen-related parenting practices. All survey items used a 4-point response scale (1=strongly disagree, 2=disagree, 3=agree, 4=strongly agree). Parent support for children’s physical activity was measured using 4 items which focused on parent facilitation of child physical activity (e.g., I take my child to places where s/he can be active, I enroll child in programs where s/he can be active), family co-participation in physical activity, and parent encouragement of child outdoor play (α = .80). Screen-related parenting was measured using 4 items assessing parental monitoring of screen time (e.g., limiting how long child plays TV, DVD, and video games; ensuring total screen time does not exceed 2 hours) (α = .74), one item assessing the frequency with which TV is on during dinner (1=never to 5=always) and one item assessing the presence of a TV in the child's bedroom (yes/no).

For food-related parenting practices, parent self-efficacy to provide healthy foods (i.e., fruit, vegetables, fat-free of low-fat milk) was assessed using 3 items (e.g., how confident are you that you can offer fruit to your child?). Response options ranged from 1=not at all confident to 5=very confident. Parent frequency of offering fruits and vegetables was measured using the mean of 2 items (i.e., How often do you offer fresh, canned or frozen fruit/vegetables to your child at meals and for snacks?). Response options ranged from 1=less than once per week to 6=three or more times per day. Additionally, parents indicated the frequency of eating from fast food restaurants like McDonald’s, Kentucky Fried Chicken, Pizza Hut, and Burger King with their child using response options ranging from 0=never to 5=every day.

#### Data analysis

All analyses were performed using SAS version 9.3 (Cary, NC). McNemar’s test was used to compare pre-post intervention differences in the percentage of children who were obese and the percentage of children with a TV in their bedroom. McNemar’s test is equivalent to a chi-square analysis and is used for dependent (i.e., time-relate) categorical variables. Paired t-tests were used to compare pre and post intervention differences in continuous measures including (a) children’s BMI z-score, physical activity, dietary intake, and screen time, and (b) food, physical activity, and screen-related parenting practices and attitudes. Analyses were performed for participants with full data (i.e., pre and post intervention) and as intent to treat analyses with baseline scores carried through to follow-up for participants who did not complete the follow-up assessment.

When significant intervention effects were identified, follow-up analyses using generalized linear models examined the effect of intervention dose. In cases where significant intervention effects were identified for multiple subdomains of a construct (e.g., all measures of dietary intake), dose analyses were limited to the most central subdomain to minimize the risk of type I error. Pre intervention levels of the outcome of interest and intervention dose were regressed onto the outcome at post intervention. Dose scores ranged from 0 to 4 and reflected the total number of intervention components to which participants reported exposure (i.e., health communication campaign, BMI letter, nutritional counseling, and the Parents Connect for Healthy Living program). Analyses were performed as intent to treat; respondents missing post intervention data received a dose score of 0.

## Results

### Sample demographic information

Because most intervention components were integrated into existing Head Start services, all families were at least minimally exposed to the intervention. Demographic characteristics of the families who agreed to participate in its evaluation (36% of all families) are outlined in Table 2. Mothers comprised the majority of the respondents, referred to collectively as parents or parent respondents. Slightly over half of the referent children were female and children were on average 3.5 years. Consistent with the general demographics of upstate New York, approximately 2/3 of participants were non-Hispanic white and 1/3 were non-White, predominantly African American. Slightly more than half of parents graduated from high school or completed some high school and approximately 70% were single and never married or part of an unmarried couple. Twenty percent of children were classified obese and 44% were overweight.

**Table 2:** **Demographic characteristics of the Communities for Healthy Living evaluation sample **[1]

**Demographic factor**	**Summary statistic**
Respondent gender (% female)	92
Child gender (% female)	55
Parent age (Mean, std)	31.13 (11.07)
Child age (Mean, std)	3.59 (1.01)
Relation of respondent to child (%)	
Mother	88
Father	6
Grandmother	6
Never speaks English at home (%)	1
Race/ethnicity (%)	
White	68
Black/African American	22
Non-Hispanic white	6
Other	4
High grade in school (%)	
Some high school	21
High school graduate	37
Some college	42
Marital status (%)	
Married	17
Divorced or separated	13
Never married/single	44
Member of unmarried couple	25
Other	1
Weight status	
Parent overweight (%)	68
Parent obese (%)	36
Child overweight (%)	44
Child obese (%)	20

[1] 154 parents completed the evaluation survey at baseline; 119 of these families completed the survey at follow-up.

### Intervention exposure

Parent respondents reported a high degree of exposure to the CHL program. Over 90% of parents recalled seeing the health communication campaign, and 85% reported reading the posters. Similarly, over 90% of parents recalled receiving the BMI letter. For the nutrition counseling sessions, 40% of parents recalled hearing about the sessions and 29% spoke with a nutrition student. Finally, 69% of parents reported hearing about the Parents Connect for Healthy Living program and 20% of respondents attended at least one program session. Overall, 80% of responding parents were exposed to 2 or more (out of a total of 4) components of the CHL program.

### Pre-post intervention differences

As shown in Table 3, significant pre-post intervention differences were identified for all child outcome categories. Compared with pre intervention, children at post intervention had marginally lower BMI z-scores and significantly lower rates of obesity. Children recorded significantly greater mins/hour in light physical activity and significantly fewer mins/day of TV viewing at post intervention compared with pre intervention; marginally greater mins/hour of moderate activity and lower mins/hour of sedentary activity at post intervention were also observed. For dietary measures, at post intervention children had significantly lower total energy intake and macronutrient intake (fat, protein, and carbohydrate) compared with pre intervention. When analyses were rerun as intent to treat analyses, results did not meaningfully differ.

**Table 3:** Pre-post intervention differences in child and parent outcomes

		**Participants with full data**					**Intent to treat**								
	**N**	**Pre Mean (std)**	**Post Mean (std)**	**Test statistic**^**1**^	**N**	**Pre Mean (std)**	**Post Mean (std)**	**Test statistic**^**1**^							
	**CHILD OUTCOMES**														
**Weight status**								
BMI z-score	136	0.79 (1.14)	0.65 (0.99)	1.69^†^	152	0.86 (1.24)	0.72 (1.12)	1.69^†^
Obese (%)	136	18.4%	13.9%	10.7^**^	152	19.7%	15.8%	10.7^**^
**Physical activity (min/hour**)								
Sedentary	57	33.2 (3.9)	32.2 (4.2)	1.83^†^	83	33.3 (4.0)	32.6 (4.2)	1.82^†^
Light physical activity	57	21.2 (2.9)	22.0 (3.3)	-2.06^*^	83	21.2 (2.9)	21.7 (3.2)	-2.04^*^
Moderate physical activity	57	4.6 (1.3)	5.0 (1.4)	-1.78^†^	83	4.7 (1.5)	4.9 (1.5)	-1.76^†^
**TV viewing time (min/day)**	93	141.9 (77.9)	71.3 (40.5)	10.0^***^	131	141.9 (77.9)	94.10 (61.16)	8.62^***^
**Dietary intake**								
Total energy (kcals)	33	1592.6 (434.3)	1403.5 (485.1)	3.40^**^	55	1513.6 (401.5)	1395.7 (423.8)	3.20^**^
Total fat (gm)	33	55.4 (17.5)	49.1 (22.7)	2.33^*^	55	50.1 (18.6)	47.3 (20.1)	2.27^*^
Total carbohydrate (gm)	33	219.6 (61.5)	194.2 (64.5)	2.69^*^	55	214.6 (57.4)	199.1 (59.4)	2.60^*^
Total protein (gm)	33	61.2 (21.1)	52.9 (20.1)	3.33^**^	55	58.1 (18.7)	52.9 (17.5)	3.15^**^
Servings of fruit	33	1.52 (1.1)	1.22 (0.7)	1.70^†^	55	1.56 (0.9)	1.37 (0.7)	1.68^†^
Servings of vegetables	33	0.79 (0.6)	0.61 (0.44)	1.56	55	0.74 (0.5)	0.63 (0.4)	1.54
Servings of grains	33	4.31 (1.9)	3.89 (1.9)	1.00	55	4.18 (1.7)	3.92 (1.6)	1.00
Servings of dairy	33	2.77 (1.3)	2.66 (1.3)	0.53	55	2.77 (1.3)	2.71 (1.3)	0.53
Servings of meat	33	3.37 (1.95)	3.13 (1.44)	1.75^†^	55	3.37 (1.95)	3.03 (1.55)	1.73^†^
	**PARENT OUTCOMES**								
**Food parenting**									
Self-efficacy to provide healthy foods^2^	99	4.61 (0.53)	4.80 (0.36)	-4.19^**^	145	4.64 (0.50)	4.78 (0.39)	-4.08^**^	
Freq. of offering fruit/veg^3^	104	4.51 (1.12)	4.69 (1.06)	-1.87^†^	145	4.43 (1.15)	4.56 (1.14)	-1.87^†^	
Freq. family eats fast food^4^	104	1.19 (0.64)	1.14 (0.61)	0.69	145	1.19 (0.61)	1.15 (0.59)	0.69	
**Physical activity parenting**									
Support for physical activity^5^	102	3.33 (0.46)	3.51 (0.44)	-3.70^**^	145	3.37 (0.51)	3.50 (0.50)	-3.36^**^	
**Screen-related parenting**									
Monitoring screen time^6^	102	3.29 (0.53)	3.27 (0.62)	0.52	145	3.34 (0.53)	3.33 (0.60)	0.52	
TV on during dinner^7^	103	1.24(1.16)	1.07 (1.12)	1.52	145	1.24 (1.17)	1.12 (1.14)	1.51	
TV in child’s bedroom	103	64%	62%	0.69	145	66%	65%	0.69	

^†^*p*<.10, ^*^p<.05, ^**^p<.01.

[1] With the exception of obesity and TV in the child’s bedroom (which were dichotomous variables), the test statistic is a t-value. For obesity and TV in the child’s bedroom, the test statistic is McNemar’s test statistic (S).

[2] Scale range: 1=low self efficacy to 5=high self efficacy; [3] Scale range: 1=less than once a week to 6 = three or more times a day; [4] Scale range: 0=never, 1=1-3 times a month to 5=every day; [5] Scale range: 1=low support to 5=high support; [6] Scale range: 1=low monitoring to 5=high monitoring; [7] Scale range: 1=never to 5=always.

Pre-post intervention differences in parenting approaches were also identified (Table 3). Compared with pre intervention, parents at post intervention reported significantly greater self-efficacy to provide healthy foods, marginally greater frequency of offering fruits and vegetables to children, and significantly greater support for children’s physical activity. No pre-post intervention differences were identified for screen-related parenting. As with child outcomes, results did not meaningfully differ when performed as intent to treat analyses.

As indicated in Table 4, significant dose effects were identified for children’s TV viewing, parents’ support of children’s physical activity, parents’ self-efficacy to provide healthy foods, and parents’ reported frequency of providing fruits and vegetables; a marginal effect of dose was identified for children’s total energy intake. In all instances, higher intervention dose predicted greater pre-post intervention improvements in the outcomes (i.e., dose predicted the outcome at post intervention controlling for pre-intervention levels).

**Table 4:** Follow-up analyses examining the effect of dose on intervention outcomes

**Outcome variable**	**Total df**	**Estimate**	**SE**	**t-value**	**p-value**
**Predictor variables**^**1**^					
Child BMI z-score (post)					
BMI z-score (pre)	133	0.71	0.058	12.09	<.0001
Dose		0.01	0.05	0.137	0. 89
Child sedentary time (post)					
Sedentary time (pre)	81	0.63	0.10	6.49	<.0001
Dose		-0.20	0.30	-0.66	0.51
Child moderate PA (post)					
Moderate PA (pre)	81	0.72	0.08	8.68	<.0001
Dose		0.08	0.09	0.86	0.39
Child TV viewing time (post)					
TV time (pre)	127	0.66	0.05	12.56	<.0001
**Dose**		**-16.59**	**2.73**	**-6.08**	**<.0001**
Child energy intake (post)					
Energy intake (pre)	49	0.83	0.10	8.67	<.0001
**Dose**		**-48.92**	**28.35**	**-1.73**	**0.09**
Parent support of PA (post)					
Support of PA (pre)	144	0.66	0.06	11.35	<.0001
**Dose**		**0.06**	**0.02**	**2.74**	**.006**
Parent self efficacy to offer healthy foods (post)	144				
Self efficacy (pre)		0.51	0.05	10.51	<.0001
**Dose**		**0.05**	**0.02**	**2.84**	**0.005**
Parent offering fruit and vegetables (post)					
Offering fruit and vegetables (pre)	144	0.72	0.05	12.89	<.0001
**Dose**		**0.10**	**0.05**	**1.89**	**0.06**

[1] The variable of interest at pre intervention and intervention dose were regressed onto the variable of interest at post intervention. Dose effects were only examined for outcomes for which significant intervention effects were identified.

## Discussion

This study introduces a novel design for family-centered childhood obesity intervention. Using CBPR principles, we worked collaboratively with low-income parents of preschool-aged children over a two-year period to develop a program that catered to families’ needs and interests, built on their strengths, responded to their constraints, and worked with them to identify and utilize assets and resources available in their communities. Positive intervention effects were identified across all child outcome domains and two out of three parenting domains. While conclusions based on these findings need to be tempered due to limitations with the study design, the consistent pattern of findings suggests that CHL, and the process by which it was developed, is a promising approach that warrants future attention in intervention design and CBPR initiatives overall.

CBPR has emerged over the past decade as a transformative research paradigm to bridge the gap between science and practice, address the challenge of program sustainability beyond research funding, and eliminate health disparities [37,38]. While CBPR has been successfully utilized to develop community [39,40], afterschool [41] and faith-based [42,43] obesity interventions, to our knowledge this is one of the first studies to use CBPR to engage low-income parents in the development, implementation, and evaluation of a family-centered obesity prevention program. At study outset, there were no preplanned elements of the intervention; all intervention components emerged through the CBPR process. As such, our *parent-centered CBPR* approach is an important departure from conventional approaches to engaging families in obesity prevention [10-12]. What’s more, its empowerment framework is a promising hybrid. Framed by FAMILI and FEM, it integrates empowerment as critical consciousness and leveraging existing resources via community and parental social capital networks.

Our parent-centered, empowerment-oriented CBPR approach has a number of advantages over more traditional models. First, it fostered parent engagement; approximately half of the CAB members attended 50% or more of the 25 CAB meetings over the 2 year study. Moreover, 5–7 parents continued to meet regularly following the intervention to plan new projects. Second, it built on pre-existing Head Start resources available to families such as BMI reporting procedures and Family Fun Days utilized for family outreach. Finally, our approach was designed to foster sustainability through capacity building (e.g., training Head Start parents as parent leaders for the Parents’ Connect program) and congruity with Head Start performance standards around parent involvement.

Despite its innovation and promising findings, results from this study are limited by the lack of a control group. The practical demands of establishing a committed and engaged CAB, conducting a comprehensive community assessment, and preparing and supporting parents as co-researchers were extensive. In short, it was not feasible in the short timeframe to establish a meaningful control group. As a result, our results need to be viewed with caution. For example, pre-post intervention differences could reflect a number of threats to internal validity [44]. Results for measures relying on parent report (e.g., child dietary intake, parent support for children’s physical activity) could be explained by parent response bias and improvements in children’s obesity risk behaviors could reflect seasonal effects. What is more, the generalizability of these findings may be limited by a selection effect with evaluation participants being disproportionately white and more likely to speak English at home than non-consenting families.

There are a number of counterarguments to possibilities. Intervention effects were also identified for objective measures not biased by parent reporting (e.g., child BMI z-scores and child physical activity) and the timeframe for this study, with pre-test during fall and posttest during winter/spring, is typically associated with changes toward less healthy behaviors in the northeastern United States [45,46]. Thus, while the magnitude of intervention effects may be overestimated in the absence of a control group, the consistent pattern of results across multiple gold standard measures and the presence of dose effects for most outcomes provide suggestive evidence of a positive impact of the CHL program.

After weighing its strengths and weaknesses, we conclude that results from this study indicate the promising nature of the CHL program and the use of parent-centered CBPR to develop family-centered interventions. Moreover, results highlight the need to develop CHL further and subject it to rigorous empirical testing. While scaling-up a CBPR-informed intervention can challenge the very essence of CBPR, our proposed scale-up strategy differs from the conventional approach. Proponents of effective translation of complex community (or in this case parent and family) interventions argue against standardization of specific intervention components across sites as it assumes that all settings have similar dynamics, cultures, and systems [47,48]. Rather, it is argued that standardization should focus on change processes, thereby leaving room for a new community to determine how they best can achieve such objectives. This approach reflects a movement away from a “best practices” to a “best processes” approach and builds on a strong theory of change [48]. With a *best process* approach in mind, expansion and further testing of the CHL program will focus on the intervention principles outlined in Table 1 such as increasing parents’ awareness of their child’s weight status, reducing myths around obesity in children, and promoting parental resource empowerment whereby they learn how to act strategically on the forces and factors impacting obesity, its determinants, and their overall well-being.

## Competing interest

The author(s) declare that they have no competing interests.

## Authors’ contributions

KD – conceived and supervised the study, analyzed the data, and led the writing; JJ – conceived and supervised the study, provided input on the analyses, and edited the manuscript for critical content; KL – led data preparation, analyzed the data, compiled the accelerometry data, and edited the manuscript for critical content; SK – led compilation of the dietary data and edited the manuscript for critical content; HL- conceived the study in conjunction with KD and JJ and edited the manuscript for critical content. All authors read and approved the final manuscript.
